# Migraine and the risk of post-traumatic stress disorder among a cohort of pregnant women

**DOI:** 10.1186/s10194-017-0775-5

**Published:** 2017-07-06

**Authors:** Lauren E. Friedman, Christina Aponte, Rigoberto Perez Hernandez, Juan Carlos Velez, Bizu Gelaye, Sixto E. Sánchez, Michelle A. Williams, B. Lee Peterlin

**Affiliations:** 1 Department of Epidemiology, Harvard T. H. Chan School of Public Health, 677 Huntington Ave, K501, Boston, MA 02115 USA; 2 Multidisciplinary International Research Training Program, Department of Epidemiology, Harvard T. H. Chan School of Public Health, Boston, MA USA; 3 Departamento de Rehabilitación, Hospital del Trabajador, Santiago, Chile; 4 Global Health and Social Medicine, Harvard Medical School, Boston, MA USA; 5 Universidad Peruana de Ciencias Aplicadas, Lima, Peru; 6 Asociación Civil PROESA, Lima, Peru; 7 Department of Neurology, Johns Hopkins School of Medicine, Baltimore, MD USA

**Keywords:** Migraine, Post-traumatic stress disorder, PTSD, Pregnancy

## Abstract

**Background:**

Individually both migraine and post-traumatic stress disorder (PTSD) prevalence estimates are higher among women. However, there is limited data on the association of migraine and PTSD in women during pregnancy.

**Methods:**

We examined the association between migraine and PTSD among women attending prenatal clinics in Peru. Migraine was characterized using the International Classification of Headache Disorders (ICHD)-III beta criteria. PTSD was assessed using the PTSD Checklist-Civilian Version (PCL-C). Multivariable logistic regression analyses were performed to estimate odds ratios (OR) and 95% confidence intervals (CI) after adjusting for confounders.

**Results:**

Of the 2922 pregnant women included, 33.5% fulfilled criteria for any migraine (migraine 12.5%; probable migraine 21.0%) and 37.4% fulfilled PTSD criteria. Even when controlling for depression, women with any migraine had almost a 2-fold increased odds of PTSD (OR: 1.97; 95% CI: 1.64–2.37) as compared to women without migraine. Specifically, women with migraine alone (i.e. excluding probable migraine) had a 2.85-fold increased odds of PTSD (95% CI: 2.18–3.74), and women with probable migraine alone had a 1.61-fold increased odds of PTSD (95% CI: 1.30–1.99) as compared to those without migraine, even after controlling for depression. In those women with both migraine and comorbid depression, the odds of PTSD in all migraine categories were even further increased as compared to those women without migraine.

**Conclusion:**

In a cohort of pregnant women, irrespective of the presence or absence of depression, the odds of PTSD is increased in those with migraine. Our findings suggest the importance of screening for PTSD, specifically in pregnant women with migraine.

**Electronic supplementary material:**

The online version of this article (doi:10.1186/s10194-017-0775-5) contains supplementary material, which is available to authorized users.

## Background

According to the National Health Interview Survey in 2011, 26.1% of women 18–44 years of age reported migraines or severe headaches in the last 3 months [1]. Migraine is more prevalent among reproductive-aged women as compared to men [2] and from early to middle adulthood as compared to younger or older individuals [1]. Migraine also often adversely affects the health of large populations [3]. Further, migraine in pregnancy is associated with an increased risk of perinatal complications including preeclampsia [4, 5], preterm delivery [6], placental abruption [7], hypertensive disorders [6], as well as cardiovascular disease and stroke [8–11].

Maternal mood and anxiety disorders have been implicated as important risk factors for migraine [12–16]. Migraine during pregnancy is associated with an increased risk of depression [17, 18] and suicidal ideation [19]. Additionally, both migraine and PTSD are more prevalent in reproductive-aged women as compared to men [2, 3]. Although increasing data supports an association between posttraumatic stress disorder (PTSD) and migraine in U.S. cohorts [3, 20, 21], no prior study has examined the risk of PTSD in pregnant women. Further there is little evidence for the association between migraine and PTSD in women from low income countries or the impact of depression on this association. To fill in these gaps in the literature, we examined the association between migraine and PTSD among a cohort of pregnant women in Lima, Peru.

## Methods

### Study population

The study population for this cross-sectional study was drawn from participants of the Pregnancy Outcomes, Maternal and Infant Study (PrOMIS) cohort. The PrOMIS cohort has been described previously [22–25]. The cohort was designed to examine maternal social and behavioral risk factors on the development of preterm birth and other adverse pregnancy outcomes among Peruvian women. The PrOMIS cohort was comprised of women attending prenatal care clinics at the Instituto Nacional Materno Perinatal (INMP) in Lima, Peru. INMP is operated by the Peruvian government and is the primary reference establishment for maternal and perinatal care. Women were eligible for inclusion if they initiated prenatal care before 16 weeks of gestation, were at least 18 years of age, and could speak and read Spanish. Pregnant women were excluded if they have mental retardation, twins, fetal malformation or a history of chronic hypertension, diabetes mellitus, sepsis or renal failure. Participants provided written informed consent. All study procedures were approved by the INMP in Lima, Peru, and the Office of Human Research Administration at the Harvard T.H. Chan School of Public Health, Boston, MA.

### Analytical population

Information was collected from participants enrolled in the PrOMIS cohort from February 2012 to March 2014. After excluding 20 women due to missing information on migraine, a total of 2922 women were included in our analysis. The excluded participants were not different from the rest of the cohort in regards to sociodemographic or lifestyle characteristics.

### Migraine assessment

Trained interviewers administered a Spanish-language questionnaire to determine migraine classification. Migraine and probable migraine status were classified based on the International Classification of Headache Disorders (ICHD)-III beta criteria [26]. Migraine was classified as participants fulfilling all 5 migraine diagnostic criteria. Probable migraine was designated if all 5 but one of the diagnostic criteria were fulfilled. Women fulfilling ICHD-III beta criteria for migraine or probable migraine when combined were classified as “any migraine.” Women not fulfilling ICHD-III beta criteria for either migraine or probable migraine were classified as non-migraineurs.

### PTSD assessment

PTSD was assessed using the PTSD Checklist-Civilian Version (PCL-C), a self-report measure with 17 items reflecting Diagnostic and Statistical Manual of Mental Disorders (DSM-IV) criteria [27]. For each item, participants were asked how bothered they were by a symptom over the past month on a 5-point Likert scale in regards to their most significant life event stressor. The total score on the PCL-C ranges from 17 to 85. Recent data from our team support that a PCL-C score of 26 or higher on the Spanish-language version is associated with an 86% sensitivity and 63% specificity in diagnosing PTSD in a Peruvian population [28]. Further, we also examined PTSD using the established cutoff score of 44 or higher [20, 29, 30].

### Other covariates

Sociodemographic characteristics and information pertaining to depression status were collected from participants through structured questionnaires. Participants’ age was classified as: 18–19, 20–29, 30–34, or ≥35 years old. Other sociodemographic covariates included: body mass index (BMI) based on self-reported pre-pregnancy weight and measured early pregnancy BMI (<18.5, 18.5–24.9, 25–29.9, >30 kg/m^2^), educational attainment (≤6, 7–12, >12 years), maternal ethnicity (Mestizos of mixed Amerindian and European descent vs. others), employment status (employed vs. not employed), marital status (married or living with partner vs. other), difficulty paying for medical care (hard vs. not very hard), difficulty paying for the very basics (hard vs. not very hard), planned pregnancy (yes vs. no), parity (nulliparous vs. multiparous), lifetime intimate partner violence (yes vs. no), childhood abuse (no abuse, physical abuse only, sexual abuse only, both physical and sexual abuse), and gestational age in weeks at the time of interview. The presence of antepartum depression was ascertained using the 9 item Spanish-language Patient Health Questionnaire-9 (PHQ-9) that has been validated in this population [31–33]. The PHQ-9 assesses depressive symptoms over the previous 14 days. The PHQ-9 score is calculated by assigning a score of 0–3 to the following response categories: “not at all,” “several days,” “more than half the days,” “nearly every day.” We defined presence of depression if participants had a PHQ-9 score ≥ 10 [34]. Lifetime intimate partner violence was ascertained through questions adapted from the Demographic Health Survey Questionnaires and Modules: Domestic Violence Module [35] and the WHO Multi-Country Study on Violence Against Women [36], respectively.

### Statistical analysis

Sociodemographic and reproductive characteristics were examined using number (percent, %) for categorical variables and mean (± standard deviation [SD]) for continuous variables. Chi-square tests were used to evaluate differences in distribution of categorical variables, and analysis of variance (ANOVA) was used to evaluate mean differences for continuous variables. Multivariable logistic regressions were used to estimate odds ratios (OR) and 95% confidence intervals (CI) of migraine in relation to PTSD.

Confounding factors were examined based on their hypothesized relationship with migraine and PTSD. Confounding was evaluated by entering potential confounders into a logistic regression model sequentially and comparing adjusted and unadjusted ORs. Final multivariable regression models included covariates that altered ORs by at least 10% or were considered *a priori* as potential confounders in the association between migraine and PTSD [37]. We examined the following covariates as potential confounders in the analyses: age, education, BMI, Mestizo ethnicity, marital status, employment, difficult paying for the basics or for medical care, parity, planned pregnancy, gestational age, childhood abuse, lifetime intimate partner violence, and depression. Prior studies have reported the comorbidity of depression and migraine [38, 39], including during pregnancy [18]. Given this, we repeated the analyses stratifying by maternal depression. In addition, some studies have used a PCL-C cutoff score of 44 to identify PTSD. Thus, we performed a sensitivity analysis using a PCL-C cutoff score of 44 or higher to diagnose PTSD. Reported *P*-values were two-sided and were statistically significant at *P* ≤ 0.05. All analyses were performed using SPSS Statistics, Version 23.0 (IBM SPSS v23.0, Armonk, NY, USA).

## Results

### Sociodemographic and reproductive characteristics

The sociodemographic and reproductive characteristics of the study population are shown in Table 1. A total of 2922 pregnant women between 18 to 35 years old (mean = 28.12 years; standard deviation (SD) = 6.31) were included in the analysis. The average gestational age at time of the interview was 9.23 weeks (SD = 3.46). The majority of the participants were married or living with a partner (81.1%), Mestizos (a race/ethnicity of mixed Amerindian and European descent; 75.2%), unemployed (53.7%), and with less than 12 years of education (59%). The prevalence of any migraine was 33.5% (migraine 12.5%; probable migraine 21.0%). Those with migraine were more likely to be unemployed, have difficulties paying for basic necessities and medical care, identify as Mestizo, and have a history of child abuse and lifetime intimate partner violence (Table 1). Of the 2922 participants, 37.4% of the participants fulfilled criteria for PTSD. Participants with PTSD were less likely to identify as Mestizo and were more likely to have difficulties paying for the basics, have difficulties paying for medical care, have a history of lifetime intimate partner violence, and suffer from depression than those without a PTSD diagnosis (Additional file 1: Table S1).

**Table 1:** Socio-demographic and reproductive characteristics of the study population according to types of migraine in Lima, Peru (*N* = 2922)

Characteristics	All participants (*N* = 2922)		No migraine (*N* = 1943)		Probable migraine (*N* = 613)		Migraine (*N* = 366)		*P*-value
	*n*	%	*n*	%	*n*	%	*n*	%	
Age (years) ^a^	28.12 ± 6.31		28.30 ± 6.31		27.76 ± 6.30		27.76 ± 6.27		0.093
Age (years)									
18–19	154	5.3	102	5.2	33	5.4	19	5.2	0.549
20–29	1642	56.2	1068	55.0	359	58.6	215	58.7	
30–34	602	20.6	411	21.2	115	18.8	76	20.8	
≥ 35	524	17.9	362	18.6	106	17.3	56	15.3	
Education (years)									
≤ 6	123	4.2	77	4.0	27	4.4	19	5.2	0.142
7–12	1599	54.8	1058	54.5	356	58.4	185	50.7	
> 12	1194	40.9	806	41.5	227	37.2	161	44.1	
Pre-pregnancy self-reported BMI									
< 18.5	30	1.2	18	1.1	9	1.7	3	1.0	0.777
18.5–24.9	1298	53.3	865	54.1	270	51.8	163	51.9	
25–29.9	851	34.9	545	34.1	189	36.3	117	37.3	
> 30	256	10.5	172	10.8	53	10.2	31	9.9	
Early pregnancy measured BMI									
< 18.5	54	1.9	26	1.4	23	3.8	5	1.4	*0.001*
18.5–24.9	1405	48.6	935	48.6	278	45.9	192	53.0	
25–29.9	1073	37.1	707	36.7	240	39.6	126	34.8	
> 30	361	12.5	257	13.4	65	10.7	39	10.8	
Mestizo ethnicity	2194	75.2	1454	74.9	491	80.1	249	68.2	*<0.001*
Married/living with a partner	2360	81.1	1566	80.9	498	81.6	296	81.1	0.927
Employed	1351	46.3	953	49.1	244	39.8	154	42.1	*<0.001*
Difficulty paying for basics									
Hard	1449	49.6	905	46.6	340	55.5	204	55.7	*<0.001*
Not very hard	1471	50.4	1036	53.4	273	44.5	162	44.3	
Difficulty paying for medical care									
Hard	1532	52.6	937	48.4	372	60.7	223	61.1	*<0.001*
Not very hard	1382	47.4	999	51.6	241	39.3	142	38.9	
Nulliparous	1425	48.9	971	50.2	289	47.2	165	45.1	0.129
Planned pregnancy	1206	41.6	823	42.6	252	41.4	131	36.1	0.067
Gestational age at interview ^a^	9.23 ± 3.46		9.28 ± 3.49		9.14 ± 3.40		9.11 ± 3.42		0.545
Intimate partner violence ^b^	1064	36.5	648	33.4	243	39.8	173	47.7	*<0.001*
Childhood abuse									
No abuse	827	28.3	590	30.4	161	26.3	76	20.8	*<0.001*
Physical only	1135	38.8	761	39.2	237	38.7	137	37.4	
Sexual only	230	7.9	155	8.0	41	6.7	34	9.3	
Both physical and sexual	730	25.0	437	22.5	174	28.4	119	32.5	
PTSD (PCL-C ≥26)	1093	37.4	585	30.1	276	45.0	232	63.4	*<0.001*
Depression (PHQ-9)	799	27.6	435	22.6	193	31.7	171	47.2	*<0.001*

Due to missing data, percentages may not add up to 100%

^a^ mean ± SD (standard deviation): How many weeks pregnant were you during your first prenatal care visit?

^b^ Lifetime intimate partner violence

For continuous variables, *P*-value was calculated using the one-way ANOVA; for categorical variables, *P*-value was calculated using the Chi-square test

*P*-values that are italicized are all <0.05

### Migraine and PTSD

A history of any migraine (migraine and probable migraine) was statistically significantly associated with increased odds of PTSD (OR = 2.50; 95% CI: 2.14–2.93) (Table 2). After adjusting for sociodemographic confounders, women who suffered from any migraine had a 2.37-fold increased odds of PTSD (95% CI: 2.02–2.79) as compared to women with no history of migraine. Further adjustment for lifetime intimate partner violence and depression status attenuated the magnitude of association but it remained significant (OR = 1.97; 95% CI: 1.64–2.37). After adjusting for sociodemographic confounders, women with migraine had a 3.81-fold increased odds of PTSD (95% CI: 3.00–4.82) as compared to non-migraineurs, and the association remained significant after adjusting for lifetime intimate partner violence and depression status (OR = 2.85; 95% CI: 2.18–3.74). Participants with probable migraine had a 1.80-fold increased odds of PTSD (95% CI: 1.49–2.18) as compared to non-migraineurs after adjusting for potential confounders. Further adjustment for depression status and lifetime intimate partner violence slightly attenuated the magnitude of association (OR = 1.61; 95% CI:1.30–1.99) (Table 1).

**Table 2:** Association between migraine and PTSD ^a^ assessed by the PCL-C during pregnancy (*N* = 2922)

Migraine Status	No PTSD (*N* = 1829)		PTSD (*N* = 1093)					
	*n*	%	*n*	%	Unadjusted OR (95% CI)	Adjusted OR (95% CI) ^b^	Adjusted OR (95% CI) ^c^	Adjusted OR (95% CI) ^d^
No migraine	1358	74.2	585	53.5	Reference	Reference	Reference	Reference
Any migraine	471	25.8	508	46.5	2.50 (2.14–2.93)	2.37 (2.02–2.79)	2.30 (1.95–2.71)	1.97 (1.64–2.37)
Types of migraine								
No migraine	1358	74.2	585	53.5	Reference	Reference	Reference	Reference
Probable migraine	337	18.4	276	25.3	1.90 (1.58–2.29)	1.80 (1.49–2.18)	1.76 (1.45–2.14)	1.61 (1.30–1.99)
Migraine	134	7.3	232	21.2	4.02 (3.18–5.08)	3.81 (3.00–4.82)	3.65 (2.86–4.66)	2.85 (2.18–3.74)

*Abbreviations*: *OR* odds ratio, *CI* confidence interval

^a^ PTSD is defined as PCL-C score ≥ 26

^b^ Adjusted for age, marital status, difficulty paying for the very basics, and difficulty paying for medical care

^c^ Adjusted for age, marital status, difficulty paying for the very basics, difficulty paying for medical care, and lifetime intimate partner violence

^d^ Adjusted for age, marital status, difficulty paying for the very basics, difficulty paying for medical care, lifetime intimate partner violence, and depression status

### Migraine and PTSD stratified by depression

Finally, we explored the association of migraine and PTSD stratified by depression status (Table 3). In a multivariable adjusted model, women with any migraine (migraine and probable migraine) but without depression had a 1.93-fold increased odds of PTSD (95% CI: 1.55–2.40) (Table 3) compared with the reference group (women without migraine or depression). Women with migraine but no depression had a 2.76-fold increased odds of PTSD (OR = 2.76; 95% CI: 1.99–3.82) after adjusting for sociodemographic confounders compared with women who had neither condition. Pregnant women with probable migraine and no depression had a 1.62-fold increased odds of PTSD (OR = 1.62, 95%CI: 1.99–3.82) compared with the reference. Compared to the reference group, participants suffering from depression and any migraine had an approximately 2.1-fold increased odds of PTSD (OR = 2.09; 95% CI: 1.49–2.92) after adjusting for potential confounders. Women with migraine or probable migraine stratified by depression had a similar increase in likelihood of PTSD compared to non-migraineurs (migraine: OR = 3.13; 95% CI: 1.91–5.11; probable migraine: OR = 1.59; 95% CI; 1.07–2.35) (Table 3).

**Table 3:** Association between migraine and PTSD ^a^ during pregnancy (*N* = 2922) stratified by depression status

Migraine Without Depression	No PTSD (*N* = 1587)		PTSD (*N* = 507)				
	*n*	%	*n*	%	Unadjusted OR (95% CI)	Adjusted OR (95% CI) ^b^	Adjusted OR (95% CI) ^c^
No migraine	1191	75.0	297	58.6	Reference	Reference	Reference
Any migraine	396	25.0	210	41.4	2.13 (1.72–2.62)	1.99 (1.61–2.46)	1.93 (1.55–2.40)
Types of migraine							
No migraine	1191	75.0	297	58.6	Reference	Reference	Reference
Probable migraine	286	18.0	129	25.4	1.81 (1.42–2.31)	1.67 (1.31–2.15)	1.62 (1.25–2.08)
Migraine	110	6.9	81	16.0	2.95 (2.16–4.04)	2.81 (2.04–3.86)	2.76 (1.99–3.82)
Migraine With Depression	No PTSD (*N* = 224)		PTSD (*N* = 575)				
	*n*	%	*n*	%	Unadjusted OR (95% CI)	Adjusted OR (95% CI) ^b^	Adjusted OR (95% CI) ^c^
No migraine	152	67.9	283	49.2	Reference	Reference	Reference
Any migraine	72	32.1	292	50.8	2.18 (1.57–3.01)	2.13 (1.53–2.96)	2.09 (1.49–2.92)
Types of migraine							
No migraine	152	67.9	283	49.2	Reference	Reference	Reference
Probable migraine	49	21.9	144	25.0	1.58 (1.08–2.31)	1.58 (1.07–2.32)	1.59 (1.07–2.35)
Migraine	23	10.3	148	25.7	3.46 (2.14–5.59)	3.29 (2.03–5.34)	3.13 (1.91–5.11)

*Abbreviations*: *OR* odds ratio, *CI* confidence interval

^a^ PTSD is defined as PCL-C score ≥ 26

^b^ Adjusted for age, marital status, difficulty paying for the very basics, and difficulty paying for medical care

^c^ Adjusted for age, marital status, difficulty paying for the very basics, difficulty paying for medical care, and lifetime intimate partner violence

The results remained similar using a PCL-C cut-off score of 44 to identify PTSD (Additional file 2: Table S2). For example, after adjusting for confounders, women who suffered from any migraine had a 3.81-fold increased odds of PTSD (95% CI: 2.76–5.26) as compared to women with no history of migraine. Further adjustment for lifetime intimate partner violence and depression status attenuated the magnitude of association, but it remained significant (OR: 2.67; 95% CI: 1.87–3.82).

## Discussion

In our cross-sectional study of pregnant Peruvian women, migraine (whether any migraine [migraine and probable migraine], migraine alone, or probable migraine alone) was associated with increased odds of PTSD. After adjusting for confounders including antepartum depression, women who reported any migraine had a 1.97-fold increased odds of PTSD (95% CI: 1.64–2.37) compared to women with no history of migraine. In a multivariable adjusted model, women with probable migraine had a 1.61-fold increased odds of PTSD (95% CI: 1.30–1.99), and women with migraine had a 2.85-fold increased odds of PTSD (95% CI: 2.18–3.74), compared to women without migraine (Table 2). In the presence of antepartum depression, women with probable migraine or migraine had increased odds of PTSD (probable migraine: OR = 1.59; 95% CI: 1.07–2.35; migraine: OR = 3.13; 95% CI: 1.91–5.11) compared to non-migraineurs (Table 3).

Previous studies have shown significant comorbidities between migraine and PTSD. However, to our knowledge, this study is the first to evaluate the association between migraine and PTSD in pregnant women. Our current findings are comparable with prior studies of adult men and non-pregnant women. In a small clinic-based study of headache patients (including migraine or tension type headache; *N* = 80), prevalence of PTSD-like symptomatology was similar to a comparison group of patients with masticatory muscle pain [40]. However, Peterlin et al. (2008) in their study of migraineurs attending an outpatient headache center demonstrated that PTSD was more frequently reported among chronic migraineurs than episodic migraineurs (42.9% vs. 9.4%, *p* = 0.0059) [20]. In a general population study in 2011, Peterlin et al. reported that those with episodic migraine had a 3- to 4- fold increased odds of PTSD as compared to those without headaches after adjusting for confounders (lifetime prevalence: OR = 3.07, 95%CI: 2.12–4.46; 12-month prevalence: OR = 4.34, 95%CI: 2.73–6.89) [41]. In a cross-sectional study in Turkey, migraine was associated with PTSD among university students (OR = 10.16, 95%CI: 3.16–32.71, *p* = 0.001) [42]. A recent study by Smitherman and Kolivas similarly found that those with migraine were almost twice as likely to fulfill diagnostic criteria for PTSD than non-migraineurs (25.7% vs. 14.2%, *p* < 0.0001). Further, compared to those without migraine, migraineurs reported more traumatic events (3.0 vs. 2.4, *p* < 0.0001) [21]. Despite differences in geographic location, population characteristics, and sociodemographics, previous findings consistently show comorbidity between migraine and PTSD.

Several potential biological and neurochemical mechanisms have been postulated for the association between migraine and PTSD. These include the biochemical markers serotonin, cortisol, and norepinephrine. Migraineurs have been shown to have imbalances of serotonin, a regulator of pain in the nervous system [43]. Serotonin levels decrease during a migraine attack, causing the trigeminal nerve to release neuropeptides and cause severe migraine pain [44]. PTSD has been previously associated with serotonin function [45, 46]. The hypothalamic–pituitary–adrenal axis and related cortisol levels have also been associated with migraine and PTSD [3, 47–49]. Additionally, decreased levels of cortisol and elevated levels of pro-inflammatory cytokines (e.g. tumor necrosis factor-alpha, interleukin-6) in patients with PTSD have been suggested to be linked to migraine [50, 51]. Videlock et al. (2008) found that norepinephrine plasma levels are lower in those with PTSD when compared to individuals without PTSD [52]. Migraine patients also may have lower levels of plasma and platelet norepinephrine [53]. Mental health during pregnancy is of particular interest given the high burden of violence in this population [24, 54]. A previous study in the same cohort found 70% of participants had a history of childhood abuse and 36.7% had a history of intimate partner violence, and their abuse history was associated with an increased risk of migraine [54]. PTSD is prevalent during pregnancy and may increase postpartum if it is not identified [55]. Although a large percentage of the population suffers from migraines, particularly those of reproductive age, the mechanisms underlying the development of migraines and PTSD have yet to be fully understood [56].

Our study has several strengths, including a large sample size and a population with a high prevalence of migraine and PTSD. However, some limitations should also be considered. First, this cross-sectional study does not establish temporal relationships between migraine and PTSD. Second, the study was conducted among low-income pregnant women in Peru; thereby, warranting caution when generalizing our study to other pregnant women. Lastly, migraine and PTSD diagnoses were established using self-reported questionnaires. Thus, we cannot exclude the possibility that PTSD and migraine status were underreported in our study. Studies that systematically use screening and confirmatory diagnostic evaluations will greatly attenuate concerns about misclassification of PTSD and migraine diagnoses in epidemiological studies [20, 21].

## Conclusions

Individually, migraine [57–59] and PTSD [60, 61] each carry a high individual, societal, and economic burden. Our study found an association between migraine and PTSD, even after adjusting for antepartum depression. Furthermore, our findings extend the body of literature on the increased risk of PTSD in those with migraine to include those with probable migraine and pregnant women. Taken together, these findings support the need for additional research on the association between migraine and PTSD, including in pregnant women, as well as the need for research evaluating potential treatment implications of this comorbidity.

## Additional files

Additional file 1: Table S1. Socio-demographic and reproductive characteristics of the study population according to PTSD ^a^ in Lima, Peru (*N* = 2922). (DOCX 36 kb)

Additional file 2: Table S2. Association between migraine and PTSD ^a^ during pregnancy (*N* = 2922). (DOCX 31 kb)

## Abbreviations

ANOVA: Analysis of variance
BMI: Body mass index
CI: Confidence interval
DSM-IV: Diagnostic and Statistical Manual of Mental Disorders
ICHD: International Classification of Headache Disorders
IL: Interleukin
INMP: Instituto Nacional Materno Perinatal
OR: Odds ratio
PCL-C: PTSD Checklist-Civilian Version
PHQ-9: Patient Health Questionnaire-9
PrOMIS: Pregnancy Outcomes, Maternal and Infant Study
PTSD: Post-traumatic stress disorder
SD: Standard deviation
