# Supplementary material for: Migraine and the risk of post-traumatic stress disorder among a cohort of pregnant women
Source: J Headache Pain. 2017 Jul 6;18(1):67. doi: 10.1186/s10194-017-0775-5 (PMC5500599; doi:10.1186/s10194-017-0775-5)
Supplement: Supplementary file 1 — Socio-demographic and reproductive characteristics of the study population according to PTSD a in Lima, Peru (N = 2922). (DOCX 36 kb) [file 10194_2017_775_MOESM1_ESM.docx]

**Supplementary Table 1: Socio-demographic and reproductive characteristics of the study population**

**according to PTSD ^a^ in Lima, Peru (N = 2,922)**

| **Characteristics** | **All participants** (N = 2,922) | |  | **No PTSD**  PCL-C < 26  (N = 1,829) | |  | **PTSD**  PCL-C ≥ 26  (N = 1,093) | |  | ***P­-*value** |
| --- | --- | --- | --- | --- | --- | --- | --- | --- | --- | --- |
|  | **n** | **%** |  | **n** | **%** |  | **n** | **%** |  |  |
| Age (years) ^a^ | 28.12 ± 6.31 | |  | 28.18 ± 6.21 | |  | 28.01 ± 6.46 | |  | 0.486 |
| Age (years) |  |  |  |  |  |  |  |  |  |  |
| 18-19 | 154 | 5.3 |  | 77 | 4.2 |  | 77 | 7.0 |  | 0.010 |
| 20-29 | 1642 | 56.2 |  | 1040 | 56.9 |  | 602 | 55.1 |  |  |
| 30-34 | 602 | 20.6 |  | 385 | 21.0 |  | 217 | 19.9 |  |  |
| ≥35 | 524 | 17.9 |  | 327 | 17.9 |  | 197 | 18.0 |  |  |
| Education (years) |  |  |  |  |  |  |  |  |  |  |
| ≤6 | 123 | 4.2 |  | 67 | 3.7 |  | 56 | 5.1 |  | 0.149 |
| 7-12 | 1599 | 54.8 |  | 1012 | 55.5 |  | 587 | 53.8 |  |  |
| >12 | 1194 | 40.9 |  | 746 | 40.9 |  | 448 | 41.1 |  |  |
| Pre-pregnancy self-reported BMI | |  |  |  |  |  |  |  |  |  |
| <18.5 | 30 | 1.2 |  | 18 | 1.2 |  | 12 | 1.3 |  | 0.153 |
| 18.5-24.9 | 1298 | 53.3 |  | 833 | 54.2 |  | 465 | 51.8 |  |  |
| 25-29.9 | 851 | 34.9 |  | 514 | 33.4 |  | 337 | 37.5 |  |  |
| >30 | 256 | 10.5 |  | 172 | 11.2 |  | 84 | 9.4 |  |  |
| Early pregnancy measured BMI |  |  |  |  |  |  |  |  |  |  |
| <18.5 | 54 | 1.9 |  | 35 | 1.9 |  | 19 | 1.8 |  | 0.523 |
| 18.5-24.9 | 1405 | 48.6 |  | 873 | 48.3 |  | 532 | 49.1 |  |  |
| 25-29.9 | 1073 | 37.1 |  | 663 | 36.7 |  | 410 | 37.8 |  |  |
| >30 | 361 | 12.5 |  | 238 | 13.2 |  | 123 | 11.3 |  |  |
| Mestizo ethnicity | 2194 | 75.2 |  | 1439 | 78.8 |  | 755 | 69.2 |  | **˂ 0.001** |
| Married/living with a partner | 2360 | 81.1 |  | 1497 | 82.1 |  | 863 | 79.4 |  | 0.070 |
| Employed | 1351 | 46.3 |  | 861 | 47.1 |  | 490 | 44.8 |  | 0.235 |
| Difficulty paying for basics |  |  |  |  |  |  |  |  |  |  |
| Hard | 1449 | 49.6 |  | 822 | 45.0 |  | 627 | 57.4 |  | **˂ 0.001** |
| Not very hard | 1471 | 50.4 |  | 1005 | 55.0 |  | 466 | 42.6 |  |  |
| Difficulty paying for medical care | |  |  |  |  |  |  |  |  |  |
| Hard | 1532 | 52.6 |  | 865 | 47.4 |  | 667 | 61.2 |  | **˂ 0.001** |
| Not very hard | 1382 | 47.4 |  | 959 | 52.6 |  | 423 | 38.8 |  |  |
| Nulliparous | 1425 | 48.9 |  | 929 | 50.9 |  | 496 | 45.5 |  | **0.005** |
| Planned pregnancy | 1206 | 41.6 |  | 787 | 43.3 |  | 419 | 38.7 |  | **0.016** |
| Gestational age at interview ^b^ | 9.23 ± 3.46 | |  | 9.26 ± 3.43 | |  | 9.19 ± 3.51 | |  | 0.588 |
| Intimate partner violence ^c^ | 1064 | 36.5 |  | 490 | 26.9 |  | 574 | 52.8 |  | **˂ 0.001** |
| Childhood abuse |  |  |  |  |  |  |  |  |  |  |
| No abuse | 827 | 28.3 |  | 666 | 36.4 |  | 161 | 14.7 |  | **˂ 0.001** |
| Physical only | 1135 | 38.8 |  | 719 | 39.3 |  | 416 | 38.1 |  |  |
| Sexual only | 230 | 7.9 |  | 137 | 7.5 |  | 93 | 8.5 |  |  |
| Both physical and sexual | 730 | 25.0 |  | 307 | 16.8 |  | 423 | 38.7 |  |  |
| Depression (PHQ-9) | 799 | 27.6 |  | 224 | 12.4 |  | 575 | 53.1 |  | **˂ 0.001** |

Due to missing data, percentages may not add up to 100%.

^a^ PTSD is defined as PCL-C score ≥ 26.

^b^ Mean ± SD (standard deviation): How many weeks pregnant were you during your first prenatal care visit?

^c^ Lifetime intimate partner violence

For continuous variables, *P*-value was calculated using the one-way ANOVA; for categorical variables, *P*-value was calculated using the Chi-square test.
