# Supplementary material for: Migraine and the risk of post-traumatic stress disorder among a cohort of pregnant women
Source: J Headache Pain. 2017 Jul 6;18(1):67. doi: 10.1186/s10194-017-0775-5 (PMC5500599; doi:10.1186/s10194-017-0775-5)
Supplement: Supplementary file 2 — Association between migraine and PTSD a during pregnancy (N = 2922). (DOCX 31 kb) [file 10194_2017_775_MOESM2_ESM.docx]

**Supplementary Table 2. Association between migraine and PTSD ^a^ during pregnancy (N = 2,922)**

| **Migraine Status** | **No PTSD**  PCL-C < 44  (N = 2,740) | |  | **PTSD**  PCL-C ≥ 44  (N = 182) | | | | | |
| --- | --- | --- | --- | --- | --- | --- | --- | --- | --- |
|  | **n** | **%** |  | **n** | **%** | **Unadjusted OR**  **(95% CI)** | **Adjusted OR**  **(95% CI) ^b^** | **Adjusted OR**  **(95% CI) ^c^** | **Adjusted OR**  **(95% CI) ^d^** |
| No migraine | 1877 | 68.5 |  | 66 | 36.3 | Reference | Reference | Reference | Reference |
| Any migraine | 863 | 31.5 |  | 116 | 63.7 | 3.82 (2.80-5.23) | 3.81 (2.76-5.26) | 3.39 (2.44-4.71) | 2.67 (1.87-3.82) |
|  |  |  |  |  |  |  |  |  |  |
| Types of migraine |  |  |  |  |  |  |  |  |  |
| No migraine | 1877 | 68.5 |  | 66 | 36.3 | Reference | Reference | Reference | Reference |
| Probable migraine | 561 | 20.5 |  | 52 | 28.6 | 2.64 (1.81-3.84) | 2.65 (1.81-3.89) | 2.45 (1.66-3.62) | 2.19 (1.43-3.35) |
| Migraine | 302 | 11.0 |  | 64 | 35.2 | 6.03 (4.19-8.68) | 5.96 (4.10-8.67) | 5.00 (3.39-7.36) | 3.28 (2.15-5.00) |

Abbreviations: OR, odds ratio; CI, confidence interval

^a^ PTSD is defined as PCL-C score ≥ 44.

^b^ Adjusted for age, marital status, difficulty paying for the very basics, and difficulty paying for medical care.

^c^ Adjusted for age, marital status, difficulty paying for the very basics, difficulty paying for medical care, and lifetime intimate partner violence.

^d^ Adjusted for age, marital status, difficulty paying for the very basics, difficulty paying for medical care, lifetime intimate partner violence, and depression status.
